# Evaluation of Bodily Pain Associated with Polycystic Ovary Syndrome: A Review of Health-Related Quality of Life and Potential Risk Factors

**DOI:** 10.3390/biomedicines10123197

**Published:** 2022-12-09

**Authors:** Kuan-Ta Lu, Yu-Cheng Ho, Chen-Lin Chang, Kuo-Chung Lan, Cheng-Chun Wu, Yu-Ting Su

**Affiliations:** 1 Department of Anesthesiology, Changhua Christian Hospital, Changhua City 50094, Taiwan; 2 School of Medicine, College of Medicine, I-Shou University, Kaohsiung City 82445, Taiwan; 3 Medical Laboratory, Medical Education and Research Center, Kaohsiung Armed Forces General Hospital, Kaohsiung City 80284, Taiwan; 4 Department of Psychiatry, Kaohsiung Armed Forces General Hospital, Kaohsiung City 80284, Taiwan; 5 Department of Obstetrics and Gynecology, Kaohsiung Chang Gung Memorial Hospital, Chang Gung University College of Medicine, Kaohsiung City 83301, Taiwan; 6 Center for Menopause and Reproductive Medicine Research, Kaohsiung Chang Gung Memorial Hospital, Chang Gung University College of Medicine, Kaohsiung City 83301, Taiwan

**Keywords:** PCOS, HRQoL, SF-36, pain, obesity, inflammation, hyperandrogenism, insulin, resistance, adipokine

## Abstract

Polycystic ovary syndrome (PCOS) is the most common reproductive disease affecting the hormone and metabolic status of women. Its associated symptoms are diverse among the patients, including hyperandrogenism, insulin resistance, anovulation, infertility, obesity, hirsutism, acne, and more. In addition, PCOS can potentially increase the risk of dysmenorrhea, endometriosis, endometrioma, and irritable bowel syndrome, which are highly related to pelvic pain and sexual difficulty. However, little known is whether PCOS exacerbates other chronic bodily pain or contributes to hyperalgesia. Health-related quality of Life (HRQoL) reflects the life satisfaction and quality derived by an individual from mental, physical, emotional, and social activities under specific conditions. In this study, we reviewed pain perception from HRQoL of PCOS patients (SF-36). The review data evidently indicated that pain perception is significantly more prevalent in patients with PCOS than in healthy controls, and obesity and infertile status could be the rationales associated with pain development. Nevertheless, underlying causes remain undetermined due to the limited information from SF-36. Furthermore, we reviewed pathophysiologic factors to pain development or exacerbation, such as the deregulation of inflammation levels, adipokines, and insulin resistance. Although current evidence of pain perception and pathophysiologic risk factors are solid in PCOS, patients’ pain perception is often ignored in clinical settings. Clinicians should note the perception and treatment of pain in PCOS patients. The correlation or causality between pain and PCOS warrants further clinical examination and basic studies, thereby providing new insights into this topic in the context of clinical diagnosis and health care.

## 1. Introduction

### 1.1. PCOS

Polycystic ovary syndrome (PCOS) is the most common form of infertility disorder; its prevalence is estimated to be approximately 4% to 20% in women of reproductive age, and it accounts for 40% of female infertility cases [1,2]. PCOS also contributes to approximately 80% of anovulatory infertility cases [3]. The clinical implications of PCOS are heterogenous across reproductive and metabolic disorders resulting from endocrine disorders such as anovulation, hyperandrogenism, obesity, insulin resistance, type 2 diabetes mellitus, hypertension, cardiovascular diseases, dyslipidemia, hirsutism, and acne [4]. Because of the hormonal anomaly associated with PCOS, the skin of patients with partial PCOS may darken and form light brown or black patches. In addition, PCOS can increase patient risk for endometrial hyperplasia and uterine cancer [5].

### 1.2. Causes of PCOS

Approximately 60% to 80% of patients with PCOS develop hyperandrogenism, androgen hyperactivation–induced ovulation disorder, adipogenesis, or insulin resistance (IR), which are the leading causes of PCOS symptoms [6]. In addition, the whole ovary of patients with PCOS may develop multiple antral follicles (AFCs) measuring 2 to 9 mm in diameter and/or an enlarged ovary measuring >10 mL. Excessive AFC development causes estrogen levels to increase, which in turn inhibits the secretion of follicle-stimulating hormones (FSHs), resulting in anovulation, prolongment of the menstrual cycle, and increased testosterone levels [7]. In addition, hyperandrogenism may increase adipogenesis and the production of serum-free fatty acids, leading to IR in PCOS pathophysiology. An excessive number of adipocytes can further release inflammation cytokines in the serum, thereby increasing IR manifestation in patients with PCOS [8]. However, the clinical symptoms of PCOS are heterogenous and may be associated with genetic disorders, environmental factors, and individual lifestyle factors, and the etiology and diagnostic criteria for PCOS are still being debated [9,10,11].

### 1.3. Health-Related Quality of Life and Pain Expression in Patients with PCOS

Health-related quality of life (HRQoL) is a general and dynamic concept that evaluates the effect of a disease or its related treatment on mental, physical, emotional, and social activities according to personal life perceptions [12]. It can represent an individual’s general status with respect to life satisfaction and quality. In the context of PCOS, PCOS-induced symptoms can considerably reduce a patient’s quality of life; therefore, PCOS-specific HRQoL assessments can be performed by administering direct patient questionnaires to obtain information that provides clinical management insights relating to the evaluation of physical, mental, and social therapies. In a self-report survey of patients with PCOS, the 36-item Short-Form Health Survey (SF-36) was used to evaluate HRQoL [13]. The SF-36 is the most used and well-established questionnaire, and it has been extensively applied to evaluate mental and physical status associated with various medical conditions [14]. It comprises eight evaluation dimensions, including physical components such as physical functioning, role limitations due to physical problems, bodily pain, and general health perceptions; it also incorporates mental components such as social functioning, mental health, and energy/vitality and role limitations due to emotional problems. SF-36 questionnaires are scored from 0 to 100, and a higher score indicates a higher quality of life.

Notably, in 2017, Martin et al. conducted semi-structured interviews and reported that pain- and discomfort-related symptoms were the most common complaints among patients with PCOS, accounting for 27.6% of the complaints made by all interviewed patients [15]. Further, HRQoL-based studies and systematic reviews are increasingly indicating that bodily pain expression is significantly elevated in patients with PCOS [15,16,17,18,19,20,21,22,23,24,25,26,27]. Although the symptoms of PCOS related to hyperandrogenism, metabolism, and reproductive function are heterogeneous and well-established, the symptoms involving bodily pain are still a topic of debate. Clinicians often overlook the pain perception of patients with PCOS, and they regard it as a PCOS symptom only during diagnosis and clinical management processes. Accordingly, understanding PCOS-specific outcomes and how PCOS symptoms affect the lives of patients is valuable to the development of clinical diagnoses and treatments related to PCOS.

## 2. Review of Studies on Pain Perception in Patients with PCOS

### 2.1. Literature Searching

To review the literature to identify the impact of PCOS on pain perception by specific HRQoL, we searched the literature from PubMed using the terms “SF-36” OR “pain” AND “polycystic ovary syndrome”. The literature was restricted to English and full text. We identified 117 studies and excluded 103 publications because of repetitions and irrelevant or not specific topics. Finally, ten studies using the questionnaire SF-36, one using SF12V2, one using semi-structured review, and two original studies matched the topic and were collected in the review (Table 1).

### 2.2. Pain-Related Results from HRQoL Surveys

Multiple studies have used the SF-36 to evaluate women with PCOS and identified the involvement of pain perception in PCOS symptoms. In 2003, Elsenbruch et al. completed the first quantitative HRQoL study in Germany; it examined how PCOS affected the bodily pain perception of women with PCOS and reported low SF-36 scores for bodily pain [16]. Their results revealed that patients with PCOS have a decreased HRQoL and that bodily pain is a major domain. Similar results were published in 2005 by Hahn et al., which were the product of an SF-36-based study of women with PCOS in Germany. Their data revealed that in addition to obesity and hirsutism, bodily pain was another key disturbance that reduced the physical quality of life of patients with PCOS [17]. An SF-36 survey of female participants with PCOS in Poland was conducted to evaluate an HRQoL program [14]. Its findings also indicated that PCOS significantly influences pain perception; specifically, patients with PCOS scored lower for pain relative to healthy controls and exhibited less favorable results for other dimensions pertaining to physical and mental quality of life [19]. Notably, the 3 aforementioned studies all reported decreased sexual satisfaction among patients with PCOS, which may be attributable to the changes in physical appearance resulting from conditions such as obesity and hirsutism [16,17]. Further research is required to clarify whether bodily pain impairs sexual intercourse in patients with PCOS.

In 2011, Li et al. conducted a meta-analysis examining the application of the SF-36 for evaluating the HRQoL of patients with PCOS; specifically, 5 studies that enrolled a total of 423 patients with PCOS and 285 healthy controls from Germany and China were examined in this meta-analysis. The results indicated that women with PCOS scored lower for all SF-36 dimensions relative to controls. For the pain domain of the SF-36, pain perception is notably greater among patients with PCOS symptoms, and patients with PCOS score significantly lower for this domain relative to controls [16,17,19,21].

In 2021, Moran-Sanchez et al. conducted a case–control study in Spain and enrolled 156 patients with PCOS and 117 age-matched healthy controls [25]. They used the Revised Life Orientation Test questionnaire to evaluate the level of optimism of patients with PCOS. A shorter pain evaluation questionnaire, specifically a modified version of the SF-36 named the SF12-V2, was administered to the participants of that study. They discovered that PCOS can impair the optimism of patients with PCOS, and more severe pain perception was also reported by patients with PCOS relative to controls. However, whether pain perception is associated with optimism level was not evaluated in the present study.

### 2.3. Effects of Obesity on Pain as Reported by HRQoL Surveys

Obesity is a common symptom of PCOS that is highly associated with hyperandrogenism and IR, and the effect of obesity and PCOS on HRQoL has been investigated. In 2006, Coffey et al. conducted an SF-36 survey in the United Kingdom to evaluate the effect of obesity on pain perception [18]. They enrolled women with PCOS from an outpatient clinic and healthy age-matched women as controls; they discovered that women with PCOS obtained lower overall SF-36 scores indicative of poorer HRQoL relative to controls and reported that the pain scores of patients with PCOS were 14% lower than those of healthy controls. In particular, they discovered that body mass index (BMI) is a crucial factor that influences pain perception; after adjusting for BMI, the pain perception of the two groups did not differ significantly. In 2010, Alvarez-Blasco et al. conducted a Spain-based SF-36 survey of women with overweight and obesity who intended to lose weight [20]. Through a comparison of patients with PCOS and their controls, Alvarez-Blasco et al. discovered that the general HRQoL mean scores of the two groups were similar and that their SF-36 pain scores were not significantly different; however, the controls obtained lower scores for a pain-related item in a Nottingham Health Profile HRQoL survey. A study conducted in Spain also reported that hyperandrogenism-related hirsutism and weight gain were the dominant factors that affected the HRQoL of women with infertility. In 2021, Naumova et al. examined patients with both infertility and PCOS, women with tubal factor infertility, and women with male factor infertility to explore the effects of these conditions on HRQoL. They reported that women with both infertility and PCOS obtained lower scores in the general SF-36 assessment and that hirsutism and weight gain were the factors most associated with HRQoL impairment. In their study, the scores for bodily pain were also lower among women with both infertility and PCOS than among those with male factor infertility [26]. These findings suggest that hyperandrogenism-related obesity is a key factor that influences the HRQoL of patients with PCOS; therefore, the influence of obesity on the pain expression of patients with PCOS warrants further studies.

### 2.4. Effects of Infertility on Pain as Reported by HRQoL Surveys

PCOS is a disease with a diverse spectrum of symptoms. In Turkey, Angin et al. enrolled women with both infertility and PCOS and women with only infertility to evaluate the effect of infertility on HRQoL [24]. Their results indicated that among the women with infertility, PCOS led to lower scores for the pain domain of the SF-36; however, the difference was nonsignificant. In a case–control study conducted in Turkey by Acmaz et al., patients with PCOS were classified according to their complaints into hirsutism, infertility, and obesity groups, and these patients were then evaluated to determine the effect of hirsutism, infertility, and obesity on their HRQoL [22]. The pain assessment results of their study revealed that patients with PCOS obtained lower pain scores in an SF-36 survey relative to their age-matched healthy controls; the results also revealed that among the patients with PCOS, infertility and hirsutism reduced pain assessment scores to a greater extent than did obesity. These data suggest that infertility and hirsutism are key symptoms that influence the disease pattern of PCOS and the pain perception of patients with PCOS.

### 2.5. Effects of Mental Status on Pain as Reported by HRQoL Surveys

In 2018, Borghi et al. performed a study in Italy to investigate the relationship of PCOS with psychological disorders and HRQoL by using the Symptom Checklist 90-R (SCL-90-R), SF-36, and State-Trait Anger Expression Inventory-2 (STAXI2). Clinical and biochemical screenings were conducted to collect blood samples obtained through serum biochemical examinations. In total, 30 patients with PCOS and 30 healthy controls were enrolled. The SF-36 questionnaire results indicated that compared to the healthy controls, the patients with PCOS obtained significantly lower scores for the physical functioning and bodily pain items of the SF-36 as well as lower scores for the bodily pain domain [23]. For the association between phenotypic/biochemical features and psychological distress, the pain perception evaluation revealed that only waist-to-hip ratio and hirsutism significantly influenced the development of psychological disorders in patients with PCOS; that is, hyperandrogenism-related biochemical anomalies were not significant factors. These HRQoL survey findings suggest that although pain perception is more prevalent in patients with PCOS than in healthy controls, the underlying causative factors of either mental or physical effects are still unclear. Obesity may be a key factor involved in pain perception; however, more basic research is required to clarify its underlying effect in this context.

### 2.6. Increased Pain Threshold among Lean Patients with PCOS

In addition to HRQoL survey-based studies on pain, researchers have also adopted pressure pain threshold (PPT) to evaluate patients’ central sensitization level, which is a condition associated with the development and maintenance of chronic pain [30]. Notably, studies have reported that lean patients with PCOS exhibited a higher PPT relative to their healthy controls [28] and that this outcome was associated with increased plasma β-endorphin levels in patients with PCOS [29]. An endogenous opioid analgesic, β-endorphin was reported to influence acute and chronic pain outcomes [31]. These data suggest that lean patients with PCOS have a higher tolerance to pain expression relative to non-lean patients with PCOS; however, whether patients with both PCOS and obesity exhibit a similar outcome is still unclear. The current literature findings appear to differ from the results obtained through HRQoL questionnaires. Although PPT has been characterized in chronic pain, increased pain expression was also identified in patients with PCOS. The outcomes of PPT in patients with PCOS require more research to clarify, especially for PCOS patients with overweight/obesity.

## 3. Potential Exacerbating Factors for Pain in Patients with PCOS

### 3.1. Effects of Low-Grade Inflammation on PCOS

Several pain subtypes can influence the daily lives of patients with PCOS. Because of the limited research on pain perception in clinical and laboratory settings, the related effects and underlying mechanisms remain unclear. Notably, PCOS manifests as a proinflammatory state in pathophysiology. Studies have reported that the levels of proinflammatory factors (e.g., proinflammatory cytokines and chemokines) were higher in the serum of patients with PCOS than in healthy controls, suggesting the effect of inflammatory status on pain perception [32,33,34,35]. A moderate increase in circulating C-reactive protein (CRP) level was also detected in patients with PCOS with or without obesity; this finding indicates that patients with PCOS develop low-grade chronic inflammation [35]. Regidor et al. reported significantly higher levels of proinflammatory lipid mediators in the serum (e.g., arachidonic acid and the arachidonic acid derivatives prostaglandins, leukotrienes, and thromboxanes) in patients with PCOS, suggesting a strong proinflammatory state in patients with PCOS relative to healthy controls [32]. Studies have reported that inflammation plays a key role in the development and maintenance of several types of pain status in the peripheral and central nervous system [36] and that the association of chronic systemic inflammation and pain sensitivity is highly involved in the development and maintenance of chronic pain [37,38]. Thus, proinflammatory status can contribute to pain perception in patients with PCOS.

### 3.2. Effects of Adipocytes in Promoting Inflammation

Researchers have attributed the association between chronic inflammatory status and PCOS to hyperandrogenism-induced abnormal adiposity and the role of both hyperandrogenism and adipocyte hypertrophy in contributing to insulin resistance [33,39,40,41,42]. The excessive deposition of adipocytes can lead to proinflammation, hypertrophy-induced hypoperfusion, compression, and hypoxia, which can activate intracellular nuclear factor kB and initiate an inflammatory reaction. This activation may further promote the synthesis and release of other inflammation mediators from adipocytes such as members of the interleukin family (IL1-β, IL-6, IL-10, and IL-18), TNF-α, TGF-β, interferon-γ, immune complement factors, sVCAM1, and MCP1. Consequently, macrophage infiltration occurs in adipocyte tissue, maintaining the inflammatory status of the patient with PCOS and impairing their adipose function [43,44,45]. In addition, the increased levels of serum-free fatty acids (i.e., ligands of toll-like receptors) in individuals with obesity can trigger immune cell reactivation [46,47,48].

Adipokines are cell-signaling proteins released by adipocytes [49]. Several adipokines with abnormalities due to atypical adipogenesis have been identified in patients with PCOS (e.g., increased levels of leptin, resistin, visfatin, RBP4, and chemerin and decreased levels of apelin, adiponectin, and omentin-1) [8,50]. Studies have reported that adipocytokines play a dual role in pain [51]. For instance, leptin can function as a proinflammatory adipokine that promotes pain perception [52,53]. Resistin is associated with post-surgery pain, migraine, and osteoarthritis, all of which can alter inflammatory conditions [54,55,56]. Visfatin is also involved in pain development in patients with osteoarthritis [57,58]. Studies have reported the analgesic effect of adiponectin in attenuating nociception in animal models of hyperalgesia [59,60]. Apelin can function as a neuropeptide, and it can ameliorate nociception in rats and mediate analgesic effects through serotonin pathways [61,62]. Consequently, the abnormal adipokine levels in patients with PCOS may become a risk factor for worsening pain perception.

### 3.3. Effects of Inflammation Cytokines and Proteins on PCOS-Related Pain

Proinflammatory cytokines are produced by reactive macrophages, and they contribute to the development of pathological pain. A study reported the involvement of TNF-α and IL-1β in inflammatory and neuropathic hyperalgesia [63], which results in an abnormal and systematic increase in pain sensitivity. The direct intraplantar injection of TNF-α also caused mechanical and thermal hyperalgesia in experimental animal models [64,65,66,67]. Further, proinflammatory cytokines and chemokines (e.g., IL-1β, TNF-α, and MCP-1) [68,69,70,71,72] may directly affect the neuronal activity in various classes of neurons in peripheral and central nerves by inciting the activity of nociceptive neurons or dorsal root ganglion (DRG) neurons. IL-6 can modulate astrocytes and microglial gliosis and control the expression of neuronal neuropeptides [73]; it is also involved in the development of neuropathic pain (including tactile allodynia and thermal hyperalgesia) in experimental rats with nerve injuries [74,75]. IL-1β can be synthesized and released by monocytes, macrophages, fibroblasts, and endothelial cells following inflammatory responses, and IL-1β was also identified in nociceptive DRG neurons [76]; thus, the intraperitoneal, intracerebroventricular, or intraplantar injection of IL-1β can trigger hyperalgesia [65,77] mechanistically by stimulating the substance P and prostaglandin E2 (PGE2) in neuronal and glial cells [78,79].

For patients with PCOS, obesity is a major symptom that has been reported to be present in approximately 50% to 80% of all patients with PCOS, and IR is a disorder involved in PCOS pathogenesis [8,80]. In one study, obesity- and IR-related syndromes were reported to be associated with chronic low-grade inflammation [34]. Researchers have revealed that hyperandrogenism is associated with inflammatory response in PCOS and that hyperglycemia (increased blood sugar level) triggered by dietary habits can promote inflammation and IR (e.g., TNF-α, IL-6, ROS, and NF-κB) in PCOS disease models [81,82,83,84]. Visceral adiposity can manifest across all weight classes in patients with PCOS [85]; however, the relationship between proinflammatory status and obesity is still being debated.

In 2011, Escobar-Morreale et al. conducted a meta-analysis study to evaluate the findings of 31 research articles regarding serum inflammatory markers in patients with PCOS; they discovered that patients with PCOS exhibited significantly higher levels of CRP relative to controls and that this difference remained significant after excluding BMI, obesity, or both. In another meta-analysis of 10 studies on IL-6 and 9 studies on TNF-α, no statistically significant differences between patients with PCOS and appropriate controls were identified [34]. Aboeldalyl et al. conducted a large-scale meta-analysis of 63 articles and verified that CRP is moderately increased in patients with PCOS, and this result was also reported in a further evaluation of nonobese women with PCOS. They further conducted a meta-analysis of articles on IL-6 expression and reported a significant difference between patients with PCOS and controls. However, no significant difference was identified in a meta-analysis of articles on TNF-α [35]. CRP is produced in the liver as an acute phase protein in response to the release of IL-6 by macrophages or adipocytes [86,87], and the serum level of IL-6 is associated with long-term health risks in women with obesity and type-2 diabetes mellitus [41,88]. Although the literature findings regarding the expression of IL-6 and TNF-α are still controversial, restrictive, and inconsistent, the status of IL-6 as a major mediator for modulating CRP synthesis and its increased levels in patients with PCOS suggest the occurrence of interactions involving IL-6 and TNF-α. Collectively, these data suggest that CRP is a key factor that indicates the chronic inflammatory status of patients with PCOS.

CRP participates in the development and maintenance of pain perception and can modulate pain sensitivity by influencing the pain threshold, thereby contributing to chronic pain [89]. An increased level of CRP is associated with the development of several pain conditions (e.g., fibromyalgia, lower back pain, pain involving opioid consumption following laparoscopic abdominal surgery, acute sciatic pain, and rheumatoid arthritis-related pain) [90,91,92,93,94]. CRP expression is also correlated with psychosocial stress, unhealthy behaviors, and sleep disorders [95]. Because patients with PCOS exhibit the pathological conditions of abnormal CRP level and chronic inflammation status, they may experience increased pain expression. However, the relationship between PCOS and these conditions requires further research to clarify.

### 3.4. Effects of Reactive Oxygen Species on PCOS-Induced Pain

An increasing body of evidence is indicating that reactive oxygen species (ROS), which are overproduced and increase the oxidative stress in patients with PCOS, are involved in PCOS pathogenesis [96,97]. Elevated ROS levels cause mitochondrial dysfunction; thus, redox potential and oxidative stress levels lead to impaired cumulus cells in the ovaries of patients with PCOS [98], thereby exacerbating disease progression. ROS are also associated with pain development and maintenance [99]; the accumulation of oxidative stress in DRG neurons is a significant inducer of hyperalgesia, which results in chronic pain [100]. Increased levels of ROS in the spinal cord can promote pain perception by reducing γ-aminobutyric-acid-mediated inhibitory neuronal transmission, which increases the expression of neuropathic pain [101]. In addition, ROS accumulation is involved in visceral pain-related amygdala plasticity; thus, the administration of ROS scavengers may provide pain relief [102]. Because patients with PCOS exhibit increased pain prevalence and ROS levels, the accumulation of ROS may be a factor that promotes pain development or maintenance.

### 3.5. Effects of IR on PCOS-Induced Pain

IR is a prevalent symptom of PCOS that results from hyperandrogenism and the increased inflammatory status derived from the presence of reactive macrophages [42]. Thus, IR-related inflammatory processes are highly correlated and may stimulate each other [103]. The key pathological features of PCOS, such as obesity, IR, hyperandrogenism, and chronic inflammation [46], are highly correlated. However, the potential of IR to induce pain is still controversial. The relationship between IR and chronic pain has been established [104], and obesity can also increase the prevalence of chronic pain [105,106]. IR is also associated with fibromyalgia syndrome [107]; this pathophysiology was reported in a population of patients with PCOS who exhibited an increased frequency of fibromyalgia [108], which is a widespread pain anomaly characterized by a decreased pain threshold. A patient with fibromyalgia may experience musculoskeletal pain accompanied by an increased frequency of disorders pertaining to sleep, fatigue, mood, and memory. Animal model and human studies have reported that acid-sensing ion channel 3 (ASIC3) is predominantly expressed in the nociceptors in the body that modulate acid-evoked pain [109]. Notably, IR can impair the expression of ASIC3, thereby inducing neuropathic pain in rat models [110,111]. In particular, metformin, a main medication for type 2 diabetes with IR and PCOS [112,113,114,115], was reported to provide pain relief for chronic pain such as rheumatoid arthritis, neuropathic pain, and fibromyalgia [116]. A report on PCOS further indicated that metformin can increase the pain threshold of lean patients with PCOS [28]. These data suggest that for patients with PCOS, IR may be a risk factor for pain expression that may further exacerbate pain development.

## 4. Summary and Discussion

This review summarized the current literature on pain perception in the context of PCOS, and the HRQoL findings obtained through the SF-36 indicate that patients with PCOS exhibit increased pain perception. Several PCOS-related pathologic factors may exacerbate pain perception, including low-grade inflammation, oxidative stress, adipogenesis, and IR. However, the questionnaire-based literature has only focused on rating pain and has not evaluated the various dimensions of pain expression such as pain type, potential causes, location, frequency, and pain scores. Thus, the association between pain expression and PCOS is still unclear. In particular, pain expression may be dynamic and diverse in patients with PCOS, and several diseases that cause bodily pain have been identified to be associated with PCOS. For instance, endometriosis and PCOS are both associated with pelvic pain [117,118,119]; they also have similar pathological causes (e.g., obesity and oxidative stress) and contribute to the increased risk of cancer development in the endometrium [120,121]. Sexual difficulties resulting from pain during intercourse are another major complaint of patients with PCOS [122,123,124], and they are typical symptoms of endometriosis. Relative to healthy individuals, patients with PCOS are more likely to develop chronic-pain-related and neuropathic-pain-related conditions such as migraine [27], fibromyalgia [107], rheumatoid arthritis [125], and irritable bowel syndrome [126,127,128]; thus, PCOS is a potential risk factor for the increased prevalence of pain-related conditions. However, the current HRQoL survey formats are limited in their ability to obtain comprehensive information regarding the pain perception of patients with PCOS. Even the survey specifically designed for patients with PCOS, namely the PCOS health-related quality-of-life questionnaire (i.e., PCOSQ), also excludes pain perception [129]. Consequently, the association between pain expression and PCOS remains unclear, and further research is required to determine whether PCOS is correlated with or causes pain expression. Furthermore, whether the pathophysiology of PCOS pertaining to low-grade inflammation, hyperandrogenism, and IR contributes to pain development and maintenance remains unclear, and further studies are necessary to clarify this topic.

Notably, an increasing body of evidence is suggesting that mental status is associated with pain perception and that this association can be bidirectional (i.e., mental status and pain perception function as risk factors and comorbidities for each other) [130,131,132,133]. Mental disorders may increase the severity of pain, whereas prolonged periods of pain contribute to the increased severity of mental disorders involving fear, stress, depression, and anxiety. Numerous HRQoL surveys have characterized mental disorders as a dominant aspect of the lives of patients with PCOS [16,17,18,20,22,23,24,25,26,134] and a factor that may affect their pain perception. However, the association between pain and mental disorders in patients with PCOS requires further clarification. A questionnaire-based survey can only provide limited information about how PCOS affects mental status. The bilateral link between physical problems and mental disorders must be further studied to identify the relevant psychological factors and improve the health care provided to patients with PCOS.

In the future, HRQoL surveys designed for patients with PCOS should include a detailed pain assessment that helps to clarify the possible causes of pain and the effects of pain on the lives of these patients, thereby helping clinicians to understand the underlying problems associated with PCOS and to develop improved strategies for health care and disease treatment. In clinics, gynecologists may be the first line who can care for and diagnose PCOS patients with abnormal pain perception—considering the susceptibility, carefully examining the source of the patient’s pain development, and evaluating the time and level of the patient’s pain perception. As early as detecting and relieving the patient’s abnormal pain perception is essential to improve the patient’s life quality. Active referral to other symptom-related clinicians is critical if the source of pain is beyond the gynecological expertise. Furthermore, basic studies should be conducted to clarify the pathological risk factors for pain in the context of PCOS; their focus should be on identifying and examining the key molecular and biological mechanisms. A model that incorporates both behavioral outcomes and molecular assessments is necessary to clarify the relationships and pathological mechanisms that link pain and PCOS. A clearer understanding of the causes of PCOS-associated symptoms can provide more insight into PCOS pathophysiology and contribute to the development of improved therapeutic approaches.

## Figures and Tables

**Table 1:** PCOS-related HRQoL and pain perception studies.

Author and Year	Research Type	Location	PCOS Diagnosis Criteria	Control	Case Number	Questionnaires	Bodily Pain in SF-36 Scores in Mean ± SD (PCOS vs. Control)	Statics	Pain Perception	Note
Elsenbruch et al., 2003 [16]	Case–control study	Germany	1990 NIH	Age-matched healthy women	PCOS *n* = 50 Healthy controls *n* = 50	SF-36, SCL-90-R, FLZ	73 ± 30 vs. 85 ± 26 (*p* < 0.05)	*	Up	
Hahn et al., 2005 [17]	Case–control study	Germany	1990 NIH	Age-matched healthy women	PCOS *n* = 120 Healthy controls *n* = 50	SF-36, SCL-90-R, VASs	74 ± 28 vs. 85 ± 26 (*p* < 0.05)	*	Up	
Drosdzol et al., 2007 [19]	Case–control study	Poland	PSE and 2003 Rotterdam	Age-matched healthy women	PCOS *n* = 50 Healthy controls *n* = 40	SF-36, ISS	64.8 ± 25.1 vs. 72.6 ± 19.8	*	Up	
Coffey et al., 2006 [18]	Case–control study	The UK	2003 Rotterdam	Age-matched healthy women	PCOS *n* = 22 Healthy controls *n* = 96	SF-36 PCOSQ	70.2 vs. 81.8	*	Up	
Alvarez-Blasco et al., 2010 [20]	Case–control study	Spain	2003 Rotterdam	Overweight and obesity age-matched women	Overweight and obesity PCOS *n* = 32 Overweight and obesity control *n* = 72	SF-36 Nottingham Health Profile (NHP)	NA	NA	No difference in SF-36; down in NHP	Obesity impairs the pain perception in PCOS patients
Li et al., 2011 [21]	Meta-analysis	Germany, Poland, China	1990 NIH 2003 Rotterdam	Age-matched healthy women	PCOS *n* = 336 Healthy controls *n* = 235	SF-36	NA	**	Up	Pain scores were lower in PCOS women
Acmaz et al., 2013 [22]	Case–control study	Turkey	2003 Rotterdam	Age-matched healthy women	PCOS *n* = 86 Healthy controls *n* = 47	SF-36, LSAS, RSES, BAI, and BDI	PCOS with hirsutism (74.5), infertility (75), obesity (79) vs. control (89)	**	Up	
Martin et al., 2017 [15]	Semi-structure interview	The US	2003 Rotterdam	NA	PCOS *n* = 20	NA	NA	NA	NA	Pain- and discomfort-related symptoms accounted for the highest proportion (27.6%) of the 735 patient expressions
Borghi et al., 2018 [23]	Case–control study	Italy	1990 NIH	Age-matched healthy women	PCOS *n* = 30 Healthy controls *n* = 30	SF-36, SCL-90R, STAXI-2	61 vs. 84	***	Up	
Angin et al., 2019 [24]	Case–control study	Turkey	2003 Rotterdam	Age-matched infertile women	49 infertile PCOS patients, 47 infertile non-PCOS patients, and 62 fertile PCOS patients	SF-36 PCOSQ	PCOS with infertility (60.9 ± 26.9) vs. infertility control 68.7 ± 21.9	Not significant	Up	
Naumova et al., 2021 [26]	Case–control study	Spain	2003 Rotterdam	Age-matched healthy women	37 infertile PCOS patients, 36 women with tubal factor infertility(TFI), and 31 women with male factor (MFI)	SF-36	PCOS(78.2 ± 22.3) vs. MFI(92.4 ± 13.2)	**	Up	
Moran-Sanchez et al., 2021 [25]	Case–control study	Spain	2003 Rotterdam	Age-matched healthy women	PCOS *n* = 156 Healthy controls *n* = 117	SF12v2 LOT-R	81.3 ± 24.6 vs. 90.5 ± 18.3	***	Up	
Kialka et al., 2016 [28]	Case–control study	Poland	2003 Rotterdam	Age-matched healthy lean women	PCOS *n* = 27 Healthy controls *n* = 18	NA	NA	NA	Down	Pain threshold is increased in lean PCOS patients
Kialka et al., 2017 [29]	Case–control study	Poland	2003 Rotterdam	Age-matched healthy lean women	PCOS *n* = 48 Healthy controls *n* = 38	NA	NA	NA	Down	Pain threshold is increased in lean PCOS patients

* *p* < 0.05, ** *p* < 0.01, *** *p* < 0.001; NA, non-appliable; SF-36, the short-form 36 questionnaire; SCL-90-R, Normative Study of Symptom Checklist 90-Revised; FLZ, Fragebogen zur Lebenszufriedenheit; VASs, visual analog scales; ISS, Index of Sexual Satisfaction; PCOSQ, Polycystic Ovary Syndrome Questionnaire; SF12v2 the 12-item Short-Form Health Survey version 2; LOT-R, Life Orientation Test-Revised.

## Data Availability

Data are contained within the article.

## References

[1] Deswal R., Narwal V., Dang A., Pundir C.S. (2020). The Prevalence of Polycystic Ovary Syndrome: A Brief Systematic Review. J. Hum. Reprod. Sci..

[2] Dennett C.C., Simon J. (2015). The role of polycystic ovary syndrome in reproductive and metabolic health: Overview and approaches for treatment. Diabetes Spectr..

[3] Balen A.H., Morley L.C., Misso M., Franks S., Legro R.S., Wijeyaratne C.N., Stener-Victorin E., Fauser B.C., Norman R.J., Teede H. (2016). The management of anovulatory infertility in women with polycystic ovary syndrome: An analysis of the evidence to support the development of global WHO guidance. Hum. Reprod. Update.

[4] Khan M.J., Ullah A., Basit S. (2019). Genetic Basis of Polycystic Ovary Syndrome (PCOS): Current Perspectives. Appl. Clin. Genet..

[5] Patel S. (2018). Polycystic ovary syndrome (PCOS), an inflammatory, systemic, lifestyle endocrinopathy. J. Steroid Biochem. Mol. Biol..

[6] Ye W., Xie T., Song Y., Zhou L. (2021). The role of androgen and its related signals in PCOS. J. Cell. Mol. Med..

[7] Szeliga A., Rudnicka E., Maciejewska-Jeske M., Kucharski M., Kostrzak A., Hajbos M., Niwczyk O., Smolarczyk R., Meczekalski B. (2022). Neuroendocrine Determinants of Polycystic Ovary Syndrome. Int. J. Environ. Res. Public Health.

[8] Chen X., Jia X., Qiao J., Guan Y., Kang J. (2013). Adipokines in reproductive function: A link between obesity and polycystic ovary syndrome. J. Mol. Endocrinol..

[9] Wang J., Wu D., Guo H., Li M. (2019). Hyperandrogenemia and insulin resistance: The chief culprit of polycystic ovary syndrome. Life Sci..

[10] Dapas M., Lin F.T.J., Nadkarni G.N., Sisk R., Legro R.S., Urbanek M., Hayes M.G., Dunaif A. (2020). Distinct subtypes of polycystic ovary syndrome with novel genetic associations: An unsupervised, phenotypic clustering analysis. PLoS Med..

[11] Teede H., Deeks A., Moran L. (2010). Polycystic ovary syndrome: A complex condition with psychological, reproductive and metabolic manifestations that impacts on health across the lifespan. BMC Med..

[12] Revicki D.A., Kleinman L., Cella D. (2014). A history of health-related quality of life outcomes in psychiatry. Dialogues Clin. Neurosci..

[13] Ware J.E., Sherbourne C.D. (1992). The MOS 36-item short-form health survey (SF-36). I. Conceptual framework and item selection. Med. Care.

[14] McHorney C.A., Ware J.E., Raczek A.E. (1993). The MOS 36-Item Short-Form Health Survey (SF-36): II. Psychometric and clinical tests of validity in measuring physical and mental health constructs. Med. Care.

[15] Martin M.L., Halling K., Eek D., Krohe M., Paty J. (2017). Understanding polycystic ovary syndrome from the patient perspective: A concept elicitation patient interview study. Health Qual. Life Outcomes.

[16] Elsenbruch S., Hahn S., Kowalsky D., Offner A.H., Schedlowski M., Mann K., Janssen O.E. (2003). Quality of life, psychosocial well-being, and sexual satisfaction in women with polycystic ovary syndrome. J. Clin. Endocrinol. Metab..

[17] Hahn S., Janssen O.E., Tan S., Pleger K., Mann K., Schedlowski M., Kimmig R., Benson S., Balamitsa E., Elsenbruch S. (2005). Clinical and psychological correlates of quality-of-life in polycystic ovary syndrome. Eur. J. Endocrinol..

[18] Coffey S., Bano G., Mason H.D. (2006). Health-related quality of life in women with polycystic ovary syndrome: A comparison with the general population using the Polycystic Ovary Syndrome Questionnaire (PCOSQ) and the Short Form-36 (SF-36). Gynecol. Endocrinol..

[19] Drosdzol A., Skrzypulec V., Mazur B., Pawlinska-Chmara R. (2007). Quality of life and marital sexual satisfaction in women with polycystic ovary syndrome. Folia Histochem. Et Cytobiol..

[20] Alvarez-Blasco F., Luque-Ramirez M., Escobar-Morreale H.F. (2010). Obesity impairs general health-related quality of life (HR-QoL) in premenopausal women to a greater extent than polycystic ovary syndrome (PCOS). Clin. Endocrinol..

[21] Li Y., Li Y., Yu Ng E.H., Stener-Victorin E., Hou L., Wu T., Han F., Wu X. (2011). Polycystic ovary syndrome is associated with negatively variable impacts on domains of health-related quality of life: Evidence from a meta-analysis. Fertil. Steril..

[22] Acmaz G., Albayrak E., Acmaz B., Baser M., Soyak M., Zararsiz G., IpekMuderris I. (2013). Level of anxiety, depression, self-esteem, social anxiety, and quality of life among the women with polycystic ovary syndrome. Sci. World J..

[23] Borghi L., Leone D., Vegni E., Galiano V., Lepadatu C., Sulpizio P., Garzia E. (2018). Psychological distress, anger and quality of life in polycystic ovary syndrome: Associations with biochemical, phenotypical andsocio-demographic factors. J. Psychosom. Obstet. Gynecol..

[24] Angin P., Yoldemir T., Atasayan K. (2019). Quality of life among infertile PCOS patients. Arch. Gynecol. Obstet..

[25] Moran-Sanchez I., Adoamnei E., Sanchez-Ferrer M.L., Prieto-Sanchez M.T., Arense-Gonzalo J.J., Carmona-Barnosi A., Hernandez-Penalver A.I., Mendiola J., Torres-Cantero A.M. (2021). Assessment of Optimism in Women with Polycystic Ovary Syndrome: A Case Control-Study. Int. J. Environ. Res. Public Health.

[26] Naumova I., Castelo-Branco C., Kasterina I., Casals G. (2021). Quality of Life in Infertile Women with Polycystic Ovary Syndrome: A Comparative Study. Reprod. Sci..

[27] Sarahian N., Noroozzadeh M., Saei Ghare Naz M., Eskandari-Roozbahani N., Mahboobifard F., Ramezani Tehrani F. (2022). Is there any association between migraine headache and polycystic ovary syndrome (PCOS)? A review article. Mol. Biol. Rep..

[28] Kialka M., Milewicz T., Sztefko K., Rogatko I., Majewska R. (2016). Metformin increases pressure pain threshold in lean women with polycystic ovary syndrome. Drug Des. Dev. Ther..

[29] Kialka M., Milewicz T., Mrozinska S., Rogatko I., Sztefko K., Majewska R. (2017). Pressure pain threshold and beta-endorphins plasma level are higher in lean polycystic ovary syndrome women. Minerva Endocrinol..

[30] Amiri M., Alavinia M., Singh M., Kumbhare D. (2021). Pressure Pain Threshold in Patients With Chronic Pain: A Systematic Review and Meta-Analysis. Am. J. Phys. Med. Rehabil..

[31] Bruehl S., Burns J.W., Chung O.Y., Chont M. (2012). What do plasma beta-endorphin levels reveal about endogenous opioid analgesic function?. Eur. J. Pain.

[32] Regidor P.A., de la Rosa X., Muller A., Mayr M., Gonzalez Santos F., Gracia Banzo R., Rizo J.M. (2022). PCOS: A Chronic Disease That Fails to Produce Adequately Specialized Pro-Resolving Lipid Mediators (SPMs). Biomedicines.

[33] Dimitriadis G.K., Kyrou I., Randeva H.S. (2016). Polycystic Ovary Syndrome as a Proinflammatory State: The Role of Adipokines. Curr. Pharm. Des..

[34] Escobar-Morreale H.F., Luque-Ramirez M., Gonzalez F. (2011). Circulating inflammatory markers in polycystic ovary syndrome: A systematic review and metaanalysis. Fertil. Steril..

[35] Aboeldalyl S., James C., Seyam E., Ibrahim E.M., Shawki H.E., Amer S. (2021). The Role of Chronic Inflammation in Polycystic Ovarian Syndrome-A Systematic Review and Meta-Analysis. Int. J. Mol. Sci..

[36] Watkins L.R., Milligan E.D., Maier S.F. (2003). Glial proinflammatory cytokines mediate exaggerated pain states: Implications for clinical pain. Adv. Exp. Med. Biol..

[37] Voscopoulos C., Lema M. (2010). When does acute pain become chronic?. Br. J. Anaesth..

[38] Baral P., Udit S., Chiu I.M. (2019). Pain and immunity: Implications for host defence. Nat. Rev. Immunol..

[39] Barber T.M., Franks S. (2013). Genetics of polycystic ovary syndrome. Polycystic Ovary Syndr..

[40] Rudnicka E., Kunicki M., Suchta K., Machura P., Grymowicz M., Smolarczyk R. (2020). Inflammatory Markers in Women with Polycystic Ovary Syndrome. BioMed Res. Int..

[41] Duleba A.J., Dokras A. (2012). Is PCOS an inflammatory process?. Fertil. Steril..

[42] Sima C., Van Dyke T.E. (2016). Therapeutic Targets for Management of Periodontitis and Diabetes. Curr. Pharm. Des..

[43] Spritzer P.M., Lecke S.B., Satler F., Morsch D.M. (2015). Adipose tissue dysfunction, adipokines, and low-grade chronic inflammation in polycystic ovary syndrome. Reproduction.

[44] Lumeng C.N., Bodzin J.L., Saltiel A.R. (2007). Obesity induces a phenotypic switch in adipose tissue macrophage polarization. J. Clin. Investig..

[45] Wang Y., Huang F. (2015). N-3 Polyunsaturated Fatty Acids and Inflammation in Obesity: Local Effect and Systemic Benefit. BioMed Res. Int..

[46] Rojas J., Chavez M., Olivar L., Rojas M., Morillo J., Mejias J., Calvo M., Bermudez V. (2014). Polycystic ovary syndrome, insulin resistance, and obesity: Navigating the pathophysiologic labyrinth. Int. J. Reprod. Med..

[47] Davis J.E., Gabler N.K., Walker-Daniels J., Spurlock M.E. (2008). Tlr-4 deficiency selectively protects against obesity induced by diets high in saturated fat. Obesity.

[48] Schaeffler A., Gross P., Buettner R., Bollheimer C., Buechler C., Neumeier M., Kopp A., Schoelmerich J., Falk W. (2009). Fatty acid-induced induction of Toll-like receptor-4/nuclear factor-kappaB pathway in adipocytes links nutritional signalling with innate immunity. Immunology.

[49] Lee M.W., Lee M., Oh K.J. (2019). Adipose Tissue-Derived Signatures for Obesity and Type 2 Diabetes: Adipokines, Batokines and MicroRNAs. J. Clin. Med..

[50] Toulis K.A., Goulis D.G., Farmakiotis D., Georgopoulos N.A., Katsikis I., Tarlatzis B.C., Papadimas I., Panidis D. (2009). Adiponectin levels in women with polycystic ovary syndrome: A systematic review and a meta-analysis. Hum. Reprod. Update.

[51] Li S., Schwartz A.V., LaValley M.P., Wang N., Desai N., Sun X., Neogi T., Nevitt M., Lewis C.E., Guermazi A. (2020). Association of Visceral Adiposity With Pain but Not Structural Osteoarthritis. Arthritis Rheumatol..

[52] Liang Y., Ma Y., Wang J., Nie L., Hou X., Wu W., Zhang X., Tian Y. (2021). Leptin Contributes to Neuropathic Pain via Extrasynaptic NMDAR-nNOS Activation. Mol. Neurobiol..

[53] Younger J., Kapphahn K., Brennan K., Sullivan S.D., Stefanick M.L. (2016). Association of Leptin with Body Pain in Women. J. Women’s Health.

[54] Hozumi J., Sumitani M., Nishizawa D., Nagashima M., Ikeda K., Abe H., Kato R., Kusakabe Y., Yamada Y., Japanese Translational Research-Cancer Pain Research Group (2019). Resistin Is a Novel Marker for Postoperative Pain Intensity. Anesth. Analg..

[55] Dehghani S., Alipoor E., Salimzadeh A., Yaseri M., Hosseini M., Feinle-Bisset C., Hosseinzadeh-Attar M.J. (2018). The effect of a garlic supplement on the pro-inflammatory adipocytokines, resistin and tumor necrosis factor-alpha, and on pain severity, in overweight or obese women with knee osteoarthritis. Phytomedicine.

[56] Rubino E., Vacca A., Govone F., Gai A., Boschi S., Zucca M., De Martino P., Gentile S., Pinessi L., Rainero I. (2017). Investigating the role of adipokines in chronic migraine. Cephalalgia.

[57] Darwish Iel S., Dessouky I.S. (2015). Does Serum Visfatin Represent a Biochemical Marker to an Experimental Peripheral Neuropathic Pain in Mice. Pharmacology.

[58] Bas S., Finckh A., Puskas G.J., Suva D., Hoffmeyer P., Gabay C., Lubbeke A. (2014). Adipokines correlate with pain in lower limb osteoarthritis: Different associations in hip and knee. Int. Orthop..

[59] Sun L., Li H., Tai L.W., Gu P., Cheung C.W. (2018). Adiponectin regulates thermal nociception in a mouse model of neuropathic pain. Br. J. Anaesth..

[60] Iannitti T., Graham A., Dolan S. (2015). Adiponectin-Mediated Analgesia and Anti-Inflammatory Effects in Rat. PLoS ONE.

[61] Turtay M.G., Karabas M., Parlakpinar H., Colak C., Sagir M. (2015). The analgesic effect of apelin-13 and its mechanism of action within the nitric oxide and serotonin pathways. Hippokratia.

[62] Xu N., Wang H., Fan L., Chen Q. (2009). Supraspinal administration of apelin-13 induces antinociception via the opioid receptor in mice. Peptides.

[63] Woolf C.J., Allchorne A., Safieh-Garabedian B., Poole S. (1997). Cytokines, nerve growth factor and inflammatory hyperalgesia: The contribution of tumour necrosis factor alpha. Br. J. Pharmacol..

[64] Cunha F.Q., Poole S., Lorenzetti B.B., Ferreira S.H. (1992). The pivotal role of tumour necrosis factor alpha in the development of inflammatory hyperalgesia. Br. J. Pharmacol..

[65] Perkins M.N., Kelly D. (1994). Interleukin-1 beta induced-desArg9bradykinin-mediated thermal hyperalgesia in the rat. Neuropharmacology.

[66] Creange A., Barlovatz-Meimon G., Gherardi R.K. (1997). Cytokines and peripheral nerve disorders. Eur. Cytokine Netw..

[67] Wagner R., Myers R.R. (1996). Endoneurial injection of TNF-alpha produces neuropathic pain behaviors. Neuroreport.

[68] Zhang J.M., Li H., Liu B., Brull S.J. (2002). Acute topical application of tumor necrosis factor alpha evokes protein kinase A-dependent responses in rat sensory neurons. J. Neurophysiol..

[69] Sorkin L.S., Xiao W.H., Wagner R., Myers R.R. (1997). Tumour necrosis factor-alpha induces ectopic activity in nociceptive primary afferent fibres. Neuroscience.

[70] White F.A., Sun J., Waters S.M., Ma C., Ren D., Ripsch M., Steflik J., Cortright D.N., Lamotte R.H., Miller R.J. (2005). Excitatory monocyte chemoattractant protein-1 signaling is up-regulated in sensory neurons after chronic compression of the dorsal root ganglion. Proc. Natl. Acad. Sci. USA.

[71] Oh S.B., Tran P.B., Gillard S.E., Hurley R.W., Hammond D.L., Miller R.J. (2001). Chemokines and glycoprotein120 produce pain hypersensitivity by directly exciting primary nociceptive neurons. J. Neurosci..

[72] Sun J.H., Yang B., Donnelly D.F., Ma C., LaMotte R.H. (2006). MCP-1 enhances excitability of nociceptive neurons in chronically compressed dorsal root ganglia. J. Neurophysiol..

[73] Klein M.A., Moller J.C., Jones L.L., Bluethmann H., Kreutzberg G.W., Raivich G. (1997). Impaired neuroglial activation in interleukin-6 deficient mice. Glia.

[74] Ramer M.S., Murphy P.G., Richardson P.M., Bisby M.A. (1998). Spinal nerve lesion-induced mechanoallodynia and adrenergic sprouting in sensory ganglia are attenuated in interleukin-6 knockout mice. Pain.

[75] Zhou Y.Q., Liu Z., Liu Z.H., Chen S.P., Li M., Shahveranov A., Ye D.W., Tian Y.K. (2016). Interleukin-6: An emerging regulator of pathological pain. J. NeuroInflamm..

[76] Copray J.C., Mantingh I., Brouwer N., Biber K., Kust B.M., Liem R.S., Huitinga I., Tilders F.J., Van Dam A.M., Boddeke H.W. (2001). Expression of interleukin-1 beta in rat dorsal root ganglia. J. Neuroimmunol..

[77] Watkins L.R., Wiertelak E.P., Goehler L.E., Smith K.P., Martin D., Maier S.F. (1994). Characterization of cytokine-induced hyperalgesia. Brain Res..

[78] Jeanjean A.P., Moussaoui S.M., Maloteaux J.M., Laduron P.M. (1995). Interleukin-1 beta induces long-term increase of axonally transported opiate receptors and substance P. Neuroscience.

[79] Schweizer A., Feige U., Fontana A., Muller K., Dinarello C.A. (1988). Interleukin-1 enhances pain reflexes. Mediation through increased prostaglandin E2 levels. Agents Actions.

[80] Goyal M., Dawood A.S. (2017). Debates Regarding Lean Patients with Polycystic Ovary Syndrome: A Narrative Review. J. Hum. Reprod. Sci..

[81] Gonzalez F., Minium J., Rote N.S., Kirwan J.P. (2005). Hyperglycemia alters tumor necrosis factor-alpha release from mononuclear cells in women with polycystic ovary syndrome. J. Clin. Endocrinol. Metab..

[82] Gonzalez F., Rote N.S., Minium J., Kirwan J.P. (2006). Reactive oxygen species-induced oxidative stress in the development of insulin resistance and hyperandrogenism in polycystic ovary syndrome. J. Clin. Endocrinol. Metab..

[83] Gonzalez F., Rote N.S., Minium J., Kirwan J.P. (2006). Increased activation of nuclear factor kappaB triggers inflammation and insulin resistance in polycystic ovary syndrome. J. Clin. Endocrinol. Metab..

[84] Gonzalez F., Rote N.S., Minium J., Kirwan J.P. (2006). In vitro evidence that hyperglycemia stimulates tumor necrosis factor-alpha release in obese women with polycystic ovary syndrome. J. Endocrinol..

[85] Carmina E., Bucchieri S., Esposito A., Del Puente A., Mansueto P., Orio F., Di Fede G., Rini G. (2007). Abdominal fat quantity and distribution in women with polycystic ovary syndrome and extent of its relation to insulin resistance. J. Clin. Endocrinol. Metab..

[86] Pepys M.B., Hirschfield G.M. (2003). C-reactive protein: A critical update. J. Clin. Investig..

[87] Lau D.C., Dhillon B., Yan H., Szmitko P.E., Verma S. (2005). Adipokines: Molecular links between obesity and atheroslcerosis. Am. J. Physiol. -Heart Circ. Physiol..

[88] Hughan K.S., Tfayli H., Warren-Ulanch J.G., Barinas-Mitchell E., Arslanian S.A. (2016). Early Biomarkers of Subclinical Atherosclerosis in Obese Adolescent Girls with Polycystic Ovary Syndrome. J. Pediatr..

[89] Afari N., Mostoufi S., Noonan C., Poeschla B., Succop A., Chopko L., Strachan E. (2011). C-reactive protein and pain sensitivity: Findings from female twins. Ann. Behav. Med..

[90] Petzke F., Clauw D.J., Ambrose K., Khine A., Gracely R.H. (2003). Increased pain sensitivity in fibromyalgia: Effects of stimulus type and mode of presentation. Pain.

[91] Giesecke T., Gracely R.H., Grant M.A., Nachemson A., Petzke F., Williams D.A., Clauw D.J. (2004). Evidence of augmented central pain processing in idiopathic chronic low back pain. Arthritis Rheum..

[92] Choi H.R., Song I.A., Oh T.K., Jeon Y.T. (2019). Perioperative C-reactive protein is associated with pain outcomes after major laparoscopic abdominal surgery: A retrospective analysis. J. Pain Res..

[93] Sturmer T., Raum E., Buchner M., Gebhardt K., Schiltenwolf M., Richter W., Brenner H. (2005). Pain and high sensitivity C reactive protein in patients with chronic low back pain and acute sciatic pain. Ann. Rheum. Dis..

[94] Lund Haheim L., Nafstad P., Olsen I., Schwarze P., Ronningen K.S. (2009). C-reactive protein variations for different chronic somatic disorders. Scand. J. Public Health.

[95] Kushner I., Rzewnicki D., Samols D. (2006). What does minor elevation of C-reactive protein signify?. Am. J. Med..

[96] Hyderali B.N., Mala K. (2015). Oxidative stress and cardiovascular complications in polycystic ovarian syndrome. Eur. J. Obstet. Gynecol. Reprod. Biol..

[97] Papalou O., Victor V.M., Diamanti-Kandarakis E. (2016). Oxidative Stress in Polycystic Ovary Syndrome. Curr. Pharm. Des..

[98] Zhao H., Zhao Y., Li T., Li M., Li J., Li R., Liu P., Yu Y., Qiao J. (2015). Metabolism alteration in follicular niche: The nexus among intermediary metabolism, mitochondrial function, and classic polycystic ovary syndrome. Free Radic. Biol. Med..

[99] Chung J.M. (2004). The role of reactive oxygen species (ROS) in persistent pain. Mol. Interv..

[100] Khasabova I.A., Khasabov S.G., Olson J.K., Uhelski M.L., Kim A.H., Albino-Ramirez A.M., Wagner C.L., Seybold V.S., Simone D.A. (2019). Pioglitazone, a PPARgamma agonist, reduces cisplatin-evoked neuropathic pain by protecting against oxidative stress. Pain.

[101] Yowtak J., Lee K.Y., Kim H.Y., Wang J., Kim H.K., Chung K., Chung J.M. (2011). Reactive oxygen species contribute to neuropathic pain by reducing spinal GABA release. Pain.

[102] Ji G., Li Z., Neugebauer V. (2015). Reactive oxygen species mediate visceral pain-related amygdala plasticity and behaviors. Pain.

[103] Chen L., Chen R., Wang H., Liang F. (2015). Mechanisms Linking Inflammation to Insulin Resistance. Int. J. Endocrinol..

[104] Zhai X., Sun C., Rong P., Li S., McCabe M.F., Wang X., Mao J., Wang S. (2016). A Correlative Relationship Between Chronic Pain and Insulin Resistance in Zucker Fatty Rats: Role of Downregulation of Insulin Receptors. J. Pain.

[105] Narouze S., Souzdalnitski D. (2015). Obesity and chronic pain: Systematic review of prevalence and implications for pain practice. Reg. Anesth. Pain Med..

[106] Malfliet A., Quiroz Marnef A., Nijs J., Clarys P., Huybrechts I., Elma O., Tumkaya Yilmaz S., Deliens T. (2021). Obesity Hurts: The Why and How of Integrating Weight Reduction With Chronic Pain Management. Phys. Ther..

[107] Pappolla M.A., Manchikanti L., Candido K.D., Grieg N., Seffinger M., Ahmed F., Fang X., Andersen C., Trescot A.M. (2021). Insulin Resistance is Associated with Central Pain in Patients with Fibromyalgia. Pain Physician.

[108] Soyupek F., Yildiz S., Akkus S., Guney M., Mungan M.T., Eris S. (2010). The Frequency of Fibromyalgia Syndrome in Patients with Polycystic Ovary Syndrome. J. Musculoskelet. Pain.

[109] Wu W.L., Cheng C.F., Sun W.H., Wong C.W., Chen C.C. (2012). Targeting ASIC3 for pain, anxiety, and insulin resistance. Pharmacol. Ther..

[110] Garcia G., Gutierrez-Lara E.J., Centurion D., Granados-Soto V., Murbartian J. (2019). Fructose-Induced Insulin Resistance as a Model of Neuropathic Pain in Rats. Neuroscience.

[111] Huang S.J., Yang W.S., Lin Y.W., Wang H.C., Chen C.C. (2008). Increase of insulin sensitivity and reversal of age-dependent glucose intolerance with inhibition of ASIC3. Biochem. Biophys. Res. Commun..

[112] American Diabetes A. (2022). Introduction: Standards of Medical Care in Diabetes-2022. Diabetes Care.

[113] Maruthur N.M., Tseng E., Hutfless S., Wilson L.M., Suarez-Cuervo C., Berger Z., Chu Y., Iyoha E., Segal J.B., Bolen S. (2016). Diabetes Medications as Monotherapy or Metformin-Based Combination Therapy for Type 2 Diabetes: A Systematic Review and Meta-analysis. Ann. Intern. Med..

[114] Lord J.M., Flight I.H., Norman R.J. (2003). Metformin in polycystic ovary syndrome: Systematic review and meta-analysis. BMJ.

[115] Naderpoor N., Shorakae S., de Courten B., Misso M.L., Moran L.J., Teede H.J. (2016). Metformin and lifestyle modification in polycystic ovary syndrome: Systematic review and meta-analysis. Hum. Reprod. Update.

[116] Baeza-Flores G.D.C., Guzman-Priego C.G., Parra-Flores L.I., Murbartian J., Torres-Lopez J.E., Granados-Soto V. (2020). Metformin: A Prospective Alternative for the Treatment of Chronic Pain. Front. Pharmacol..

[117] Singh K.B., Patel Y.C., Wortsman J. (1989). Coexistence of polycystic ovary syndrome and pelvic endometriosis. Obstet. Gynecol..

[118] Kichukova D. (1996). Polycystic ovaries in association with pelvic endometriosis in infertile women diagnosed by laparoscopy. Folia Med..

[119] Holoch K.J., Savaris R.F., Forstein D.A., Miller P.B., Higdon H.L., Likes C.E., Lessey B.A. (2014). Coexistence of Polycystic Ovary Syndrome and Endometriosis in Women with Infertility. J. Endometr. Pelvic Pain Disord..

[120] Ding D.C., Chen W., Wang J.H., Lin S.Z. (2018). Association between polycystic ovarian syndrome and endometrial, ovarian, and breast cancer: A population-based cohort study in Taiwan. Medicine.

[121] Lu J., Wang Z., Cao J., Chen Y., Dong Y. (2018). A novel and compact review on the role of oxidative stress in female reproduction. Reprod. Biol. Endocrinol..

[122] Loh H.H., Yee A., Loh H.S., Kanagasundram S., Francis B., Lim L.L. (2020). Sexual dysfunction in polycystic ovary syndrome: A systematic review and meta-analysis. Hormones.

[123] Nohr E.A., Hansen A.B., Andersen M.S., Hjorth S. (2022). Sexual health in parous women with a history of polycystic ovary syndrome: A national cross-sectional study in Denmark. Int. J. Gynecol. Obstet..

[124] Taghavi S.A., Bazarganipour F., Hugh-Jones S., Hosseini N. (2015). Health-related quality of life in Iranian women with polycystic ovary syndrome: A qualitative study. BMC Women’s Health.

[125] Sharmeen S., Nomani H., Taub E., Carlson H., Yao Q. (2021). Polycystic ovary syndrome: Epidemiologic assessment of prevalence of systemic rheumatic and autoimmune diseases. Clin. Rheumatol..

[126] Tseng P.H., Chiu H.M., Tu C.H., Wu M.S., Ho H.N., Chen M.J. (2021). Obesity Exacerbates Irritable Bowel Syndrome-Related Sleep and Psychiatric Disorders in Women With Polycystic Ovary Syndrome. Front. Endocrinol..

[127] Kaluzna M., Kompf P., Wachowiak-Ochmanska K., Moczko J., Krolczyk A., Janicki A., Szapel K., Grzymislawski M., Ruchala M., Ziemnicka K. (2022). Are patients with polycystic ovary syndrome more prone to irritable bowel syndrome?. Endocr. Connect..

[128] Bazarganipour F., Taghavi S.A., Asemi Z., Allan H., Khashavi Z., Safarzadeh T., Pourchangiz S., Zare F., Ghasemi S., Karimi Z. (2020). The impact of irritable bowel syndrome on health-related quality of life in women with polycystic ovary syndrome. Health Qual. Life Outcomes.

[129] Cronin L., Guyatt G., Griffith L., Wong E., Azziz R., Futterweit W., Cook D., Dunaif A. (1998). Development of a health-related quality-of-life questionnaire (PCOSQ) for women with polycystic ovary syndrome (PCOS). J. Clin. Endocrinol. Metab..

[130] Michaelides A., Zis P. (2019). Depression, anxiety and acute pain: Links and management challenges. Postgrad. Med..

[131] Bair M.J., Robinson R.L., Katon W., Kroenke K. (2003). Depression and pain comorbidity: A literature review. Arch. Intern. Med..

[132] Sykioti P., Zis P., Vadalouca A., Siafaka I., Argyra E., Bouhassira D., Stavropoulou E., Karandreas N. (2015). Validation of the Greek Version of the DN4 Diagnostic Questionnaire for Neuropathic Pain. Pain Pract..

[133] Varrassi G., Fusco M., Coaccioli S., Paladini A. (2015). Chronic pain and neurodegenerative processes in elderly people. Pain Pract..

[134] Patten R.K., Pascoe M.C., Moreno-Asso A., Boyle R.A., Stepto N.K., Parker A.G. (2021). Effectiveness of exercise interventions on mental health and health-related quality of life in women with polycystic ovary syndrome: A systematic review. BMC Public Health.

